# Adipose Tissue and Biological Factors. Possible Link between Lymphatic System Dysfunction and Obesity

**DOI:** 10.3390/metabo11090617

**Published:** 2021-09-11

**Authors:** Klaudia Antoniak, Rita Hansdorfer-Korzon, Małgorzata Mrugacz, Katarzyna Zorena

**Affiliations:** 1 Department of Immunobiology and Environment Microbiology, Medical University of Gdańsk, Dębinki 7, 80-211 Gdańsk, Poland; klaudia.antoniak@gumed.edu.pl; 2 Department of Physical Therapy, Medical University of Gdańsk, Dębinki 7, 80-211 Gdańsk, Poland; rita.hansdorfer-korzon@gumed.edu.pl; 3 Department of Ophthalmology and Eye Rehabilitation, Medical University of Bialystok, Kilinskiego 1, 15-089 Białystok, Poland; malgorzata.mrugacz@umb.edu.pl

**Keywords:** adipose tissue, obesity, biological factors, lymph-angiogenesis, lymphatic contractile and lymphatic vascular permeability, manual lymphatic drainage

## Abstract

The World Health Organization (WHO) has recognised obesity as one of the top ten threats to human health. Obesity is not only a state of abnormally increased adipose tissue in the body, but also of an increased release of biologically active metabolites. Moreover, obesity predisposes the development of metabolic syndrome and increases the incidence of type 2 diabetes (T2DM), increases the risk of developing insulin resistance, atherosclerosis, ischemic heart disease, polycystic ovary syndrome, hypertension and cancer. The lymphatic system is a one-directional network of thin-walled capillaries and larger vessels covered by a continuous layer of endothelial cells that provides a unidirectional conduit to return filtered arterial and tissue metabolites towards the venous circulation. Recent studies have shown that obesity can markedly impair lymphatic function. Conversely, dysfunction in the lymphatic system may also be involved in the pathogenesis of obesity. This review highlights the important findings regarding obesity related to lymphatic system dysfunction, including clinical implications and experimental studies. Moreover, we present the role of biological factors in the pathophysiology of the lymphatic system and we propose the possibility of a therapy supporting the function of the lymphatic system in the course of obesity.

## 1. Adipose Tissue—Structure and Functions

The 21st century epidemic of obesity has led scientists to pay more attention to adipose tissue in recent years. Until recently, adipose tissue was considered a place of energy storage [1]. Currently, it is known that adipose tissue has an insulating function and, located just under the skin and around larger organs, naturally protects these against mechanical trauma. Moreover, it is an important energy store, activated at times of increased energy demand of the body [2].

The main ingredients of adipose tissue are cells called adipocytes, although it also contains preadipocytes, fibroblasts, leukocytes, monocytes, macrophages, endothelial cells, and a subpopulation of stem cells called SVF (stromal vascular fraction) cells [3,4].

Adipose tissue can be divided into white, brown, beige and pink tissue [4,5,6]. From a physiological point of view, all four types of fat cell have endocrine properties. White adipocytes form white adipose tissue (WAT), which stores energy. Each adipocyte of white adipose tissue is filled with one large drop of triglycerides, which is the largest part of their cell volume [5].

Both the cell organelles and the cytoplasm are located peripherally. White adipose tissue is less vascularised and contains less extracellular matrix compared to brown adipose tissue, which results from the different functions of both types of adipose tissue [6]. Brown adipocytes are the main type of parenchymal cells found in brown adipose tissue (BAT). These cells are also called multilocular adipocytes. The brown adipocyte burns lipids to produce heat: its multilocularity maximises the cytoplasmic–lipid interface, making large amounts of fatty acids available quickly for mitochondrial uncoupling and consequently thermogenesis [3,4]. Experimental studies from recent decades have shown that adipose tissue has the possibility of cooperation between WAT and BAT. The main function of the adipose organ appears to be the division of energy derived from nutrients into two different pathways: WAT for metabolism and BAT for thermogenesis [6]. Moreover, each tissue is able to cooperate and reversibly transform WAT ↔ BAT [3,4]. In fact, during chronic exposure to cold, WAT differentiates to BAT, and during a chronic positive energy balance, BAT differentiates to WAT. In studies with R26R double transgenic mice (ROSA26 reporter), white-to-pink adipocyte trans-differentiation (‘pinking’) was demonstrated during pregnancy (days 17–18). On the other hand, in the post-lactation period, the mammary gland changes rapidly, and the pink adipocytes are converted into white [4].

The functions of adipose tissue, present locally within the organs, muscles, epicardium, and inside the vessels and kidneys, are different according to location. Visceral adipose tissue (VAT) is responsible for local and generalised inflammation, for insulin resistance in muscles, for local inflammation and chemotaxis in the epicardium [7,8]. VAT affects atherosclerosis and arterial hypertension, and increases intravascular pressure within the kidneys [9]. The above-mentioned functions of VAT are performed by the production and secretion of numerous cytokines and adipokines, including leptin, adiponectin, resistin, visfatin, apelin, vaspin, chemerin, tumour necrosis factor α (TNF-α), interleukin 6 (IL-6), monocyte chemotactic protein-1 (MCP-1), plasminogen activator inhibitor-1 (PAI-1), retinol-binding protein (RBP-4), omentin and progranulin [3,5,10]. In the light of contemporary research, it is known that adipose tissue functions as an endocrine organ and in physiological conditions aims to maintain the activity of the immune system as well as the homeostasis of pro- and anti-inflammatory factors [5,11,12,13,14,15,16,17,18]. In the case of organism dysregulation, e.g., by excessive growth of adipose tissue, it ceases to fulfil its homeostatic functions, as well as ceasing to maintain the stability of the internal environment, which in turn may lead to the development of metabolic diseases and chronic vascular complications [7,8,9,12]. 

## 2. Adipose Tissue Immune Cells and Biological Factors

Adipose tissue is a complex structure responsible for fat storage, biological factors and metabolites, with systemic actions [3,5,7]. Expansion of adipose tissue is accompanied by the infiltration of different types of immune cells (macrophage, neutrophils, lymphocytes, etc.), which induces a state of low-grade, chronic inflammation and metabolic dysregulation [11,12,13,14,15,16,17,18,19,20,21,22,23,24,25,26,27,28,29,30,31,32,33,34,35,36,37,38,39,40,41,42,43,44]. Even though the exact mechanism of this low-grade inflammation is not fully understood, there is clear evidence that adipose tissue-infiltrating macrophages play a significant role in the pro-inflammatory state and in dysregulated metabolism adipose tissue [11,12,13,14,15,16,17,18]. 

### 2.1. Macrophages

Macrophages are the most common cells of the immune system, accounting for 40–60% of all immune cells in adipose tissue [11]. It has been shown that the number of macrophages in adipose tissue increases successively after the first week of using a high-fat tissue diet. [11,12]. Moreover, in obesity, apart from the increase in the number of adipose tissue macrophages, their polarization is observed, as M2 anti-inflammatory macrophages turn into a pro-inflammatory phenotype—M1 macrophages [13]. M1 macrophages are activated by cytokines produced by type 1 T helper cells (Th1), which release Interferon-γ (IFN-γ) [14]. Activated M1 macrophages are responsible for the secretion of pro-inflammatory cytokines such as TNF-α, interleukin 1β (IL-1β) and Interleukin 6 (IL-6), which results in the activation of induced nitric oxide synthase (iNOS) and nitric oxide (NO) [12,14,15,16]. Moreover, M1 macrophages produce pro-inflammatory cytokines: interleukin 12 (IL-12) and interleukin 23 (IL-23), with a simultaneous decreased synthesis of an anti-inflammatory cytokine: interleukin 10 (IL-10) [17]. It has been shown that M1 macrophages are the main source of pro-inflammatory cytokines, which is associated not only with obesity, but also with the development of insulin resistance (IR) [18].

### 2.2. Neutrophils

Other types of innate immune cells in the visceral and perivascular adipose tissue include neutrophils, which constitute approximately 2% of the immune cell fraction [19]. In the early stages of obesity, neutrophils penetrate adipose tissue, where they produce chemokines and cytokines, thus promoting macrophage infiltration [20]. The accumulation of neutrophils stimulates inflammation in adipose tissue primarily through the production of TNF-α, MCP-1 and IL-1β [20,21,22].

### 2.3. Mast Cells

Studies in mice have shown that another consequence of obesity is the increase in the number of mast cells in adipose tissue [23]. Mast cells respond to the microenvironment by releasing preformed content of granules (histamine, heparin, tryptase and chymase) or by releasing pro-inflammatory cytokines such as interleukin 1 (IL-1), interleukin 3 (IL-3), interleukin 5 (IL-5), (IL-6), interleukin 8 (IL-8) and TNF-α [23,24]. At the same time, the increased number of mast cells additionally mediates the stimulation of the influx of M1 macrophages [23]. Mast cells also stimulate the release of cathepsin, which induces proteolysis of the intercellular substance and angiogenesis, thus causing the reconstruction of adipose tissue in obese people [25].

### 2.4. T Lymphocytes

Lymphocytes are the main type of immune cell found in the lymph, which is where they get their name. Lymphocytes include T cells (for cell-mediated, cytotoxic adaptive immunity) and B cells (for humoral, antibody-driven adaptive immunity) [26]. So far, the basis for the classification of T lymphocytes has been the presence of the T-cell receptor (TCR), class I histocompatibility antigens (MHC I) and differentiation receptors (CD) [26,27]. The current division of T lymphocytes is based not only on surface receptors, but mainly on the production of substances and their functions, which has led to the differentiation of several types of T lymphocytes [27,28,29,30,31,32,33].

#### 2.4.1. Regulatory T Cells

Regulatory T (Treg) cells show a high expression of an anti-inflammatory cytokine: IL-10, which inhibits macrophage migration and the change of macrophage phenotype towards anti-inflammatory M2 type [27]. Both in murine models and in obese patients, a decrease in the number of Treg cells in visceral adipose tissue was found [28]. It has been observed that the decrease in the number of Treg cells in mice increases the concentration of insulin and pro-inflammatory cytokines such as IL-1β, IL-6, IL-8, TNF-α, and macrophage inflammatory protein-1α (MIP-1α) [27,28,29]. These data suggest that the increase in the number of Treg cells may reduce M1 macrophage influx and contribute to the reduction of adipose tissue inflammation, playing a particular role in improving insulin sensitivity [29]. In adipose tissue, apart from Treg cells, there are also other populations of T cells that influence the course of inflammation [27,29].

#### 2.4.2. CD4^+^ T Cells

Naive CD4^+^ T cells settle in secondary lymphoid organs (such as the spleen and lymphocytes) and nonlymphoid organs (such as adipose tissue) after developing in the thymus [27]. CD4^+^ T cells can be divided into three groups of helper cell: T-helper type 1 (Th1), T-helper type 2 (Th2) and T-helper type 17 (Th17) [27,29,30]. T cells that promote the development of insulin resistance, atherosclerosis and hypertension mainly include Th1 cells producing IFN-γ, Th17 cells producing TNF-α and interleukin 17 (IL-17) [29]. The decrease in the concentration of Th2 cells observed in obesity results in a decrease in the concentration of anti-inflammatory cytokines, such as interleukin 4 (IL-4), interleukin 5 (IL-5), interleukin 10 (IL-10) and interleukin 13 (IL-13) [31].

#### 2.4.3. Cytotoxic CD8^+^ T Cells

CD8^+^ Tc lymphocytes, also referred to as CTL (cytotoxic T lymphocytes), destroy cells that recognize foreign MHC class I molecules or antigens associated with their own MHC class I molecules [32]. So far, both experimental and clinical studies have shown that CD8^+^ lymphocytes can increase the production of pro-inflammatory factors, e.g., TNF-α, interleukin 2 (IL-2), IFN-γ and RANTES chemokines, contributing to inflammation in the course of obesity [33,34]. In studies in obese mice, an interaction was detected between CD8^+^ T cells, macrophages and adipose tissue. It has been shown that infiltration of adipose tissue by CD8^+^ T cells is preceded by macrophage accumulation, production of inflammatory factors, and disclosure of inflammation. Conversely, a reduction in the number of CD8^+^ T lymphocytes reduced the influx of macrophages, decreased adipose tissue inflammation and improved insulin sensitivity [34].

### 2.5. B Lymphocytes

**Figure 1:**
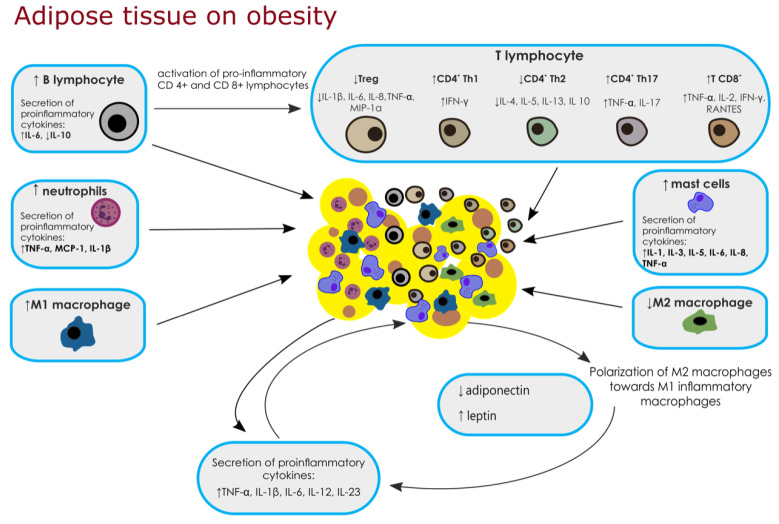
Diagram showing biological factors in adipose tissue in obesity. Description in the text. Abbreviations: ↑ — increase, ↓ — decrease, TNF-α (tumor necrosis factor-α); IL-1β (Interleukin-1β); IL-6 (Interleukin-6); IL-12 (Interleukin-12); IL-23 (Interleukin-23); iNOS (induced nitric oxide synthase); NO (nitric oxide); IL-10 (Interleukin-10); MCP-1 (Monocyte Chemoattractant Protein-1); IL-1 (Interleukin-1); IL-3 (Interleukin-3); IL-5 (Interleukin-5); IL-8 (Interleukin-8); Treg (regulatory T cells); MIP-1α (macrophage inflammatory protein-1α); Th1 (T-helper-type 1); IFN-γ (Interferon-γ); Th2 (T-helper-type 2); IL-4(Interleukin-4); IL-13(Interleukin-13); Th17 (T-helper-type 17); IL-17 (Interleukin-17); IL-2(Interleukin-2); plasminogen activator inhibitor-1 (PAI-1).

B lymphocytes can be divided into two classes, B1 and B2 cells [8,35,36,37]. In murine models, B2 cells in VAT produce pathogenic IgG, while network B1 cells produce “natural antibody” IgM with anti-inflammatory properties [8]. The authors found that a high-fat diet in mice may affect VAT through augmentation of class-switched mature B cells [8]. At the same time, an increase in serum of pro-inflammatory adipose-specific IgG2c has been reported, while when B cells were transferred into B^null^ mice (μ heavy chain knock-out), there was impaired glucose tolerance and elevated levels of fasting insulin [8]. B lymphocytes can activate pro-inflammatory CD8^+^ and CD4^+^ T cells and induce a change in the phenotype of M2 macrophages [34]. Preliminary studies have shown that mice fed a high-fat diet secrete more pro-inflammatory cytokines such as IL-6 and TNF-α than B cells of mice fed a normal fat diet [35]. On the other hand, in obese patients, B-cell studies showed an increase in IL-6 production and a decrease in IL-10 production [36,37]. Figure 1 shows possible changes in adipose tissue under the influence of biological factors.

## 3. Saturated Fatty Acids

Previous studies have shown that saturated fatty acids (SFA) may play different roles in obesity and type 2 diabetes (T2D), as well as cardiovascular diseases [18,35]. In the European Prospective Investigation into Cancer and Nutrition Study (EPIC) and the Norfolk Prospective Study it has been seen that, even-chain saturated fatty acid concentrations were associated with an increased risk of coronary heart disease and T2D, whereas odd-chain saturated fatty acid concentrations were associated with a decreased risk [38]. In a cross-sectional study of 484 Japanese participants, of men with an average age 44.6 years, and women with an average age of 43.1 years, the authors examined the association of saturated fatty acids in serum phospholipids with circulating levels of adipokines [39]. Higher levels of even-chain saturated fatty acids (14:0 myristic, 16:0 palmitic and 18:0 stearic acids) were associated with higher levels of resistin and lower levels of adiponectin. By contrast, odd-chain saturated fatty acids (15:0 pentadecanoic and 17:0 heptadecanoic acids) showed inverse associations with leptin and PAI-1. Visfatin was positively associated with both even- and odd-chain saturated fatty acids. In conclusion, the authors suggest that odd-chain saturated fatty acids are associated with a favourable adipokine profile, whereas even-chain saturated fatty acids are associated with an unfavourable profile [39].

Saturated fatty acids promote the inflammatory activation of macrophages, also activating the pathways of nuclear factor kappa-light-chain-enhancer of activated B cells (NF-κB) i c-Jun N-terminal kinases (JNK) [40,41]. After these pathways are stimulated, an increase in the secretion of chemokines such as MCP-1 and TNF-α takes place, which, when released from adipocytes, cause inflammatory macrophage infiltration [40]. Additionally, in obesity, adipose tissue increases its volume in response to excessive energy supply by increasing the number of fat cells (hyperplasia) and increasing the size of fat cells (hypertrophy) [42]. Increased adipocyte size is characterised by a higher adipocyte death rate and macrophage recruitment [42,43]. On the other hand, overgrown adipocytes show altered secretion of chemo-attractants and immune proteins, which may also favour pro-inflammatory macrophage infiltration [44]. A summary of the role of adipose tissue is presented in Table 1.

## 4. Adipose Tissue, Obesity and the Autonomic Nervous System

An important system involved in the regulation of adipose tissue functions and obesity-related processes is the autonomic nervous system (ANS) [45,46,47,48,49]. The sympathetic system is commonly known as “fight or flight” and the parasympathetic part as “rest and digest” [45,48]. Therefore, an imbalance of the sympathetic (SNS) and parasympathetic (PNS) systems of the autonomic nervous system may contribute to the dysfunction of systems (including the lymphatic system), organs, e.g., heart, pancreas, and consequently the development of many diseases [45,46,47,48,49,50,51,52]. Over recent decades, evidence has accumulated that the ANS plays a key role in inflammation [46], obesity pathogenesis, diabetes and chronic vascular complications [46,47,48,49,50,51,52,53]. 

Overweight and obesity are associated with increased SNS activity and decreased PNS function [54,55]. Many experimental and clinical studies have shown that SNS hyperactivity is a characteristic of obesity and may possibly increase the risk of developing metabolic syndrome [56]. This is confirmed by previous findings indicating an increase in serum norepinephrine indices, as well as renal and cardiac indices, noradrenaline spill-over rate (NA-SR), sympathetic nerve activity (SNA) and heart rate variability (HRV) in people with abnormal body weight [57,58]. The first reports indicate that SNS hyperactivity is a compensatory response aimed at increasing resting energy expenditure in order to reduce body weight [59]. This hypothesis was revised in later studies, indicating chronic SNS activity as a factor causing weight gain caused by decreased beta-adrenoceptor activity [60]. Chronic SNS hyperactivity occurs primarily in central obesity [61]. Recent studies show increased sympathetic activity in obese patients, especially in the vascular system of muscles and kidneys, which may contribute to an increased cardiovascular risk [62]. Increased sympathetic activity may also reduce insulin sensitivity, determining the vicious cycle responsible for hypertension and the development of metabolic syndrome [47,63].

Moreover, the ANS has been shown to play a major role in the regulation of food intake, including satiety signals and energy expenditure [50,51,52]. Additionally, afferent vagal pathways, as part of the ANS, are indicated as the most important link between the intestines and the brain and as a factor interacting with intestinal hormones [53]. Conversely, obesity may induce changes in the sympathetic regulation of the functions of the cardiovascular system, thus promoting the development of complications and increasing the risk of cardiovascular sequelae [64]. The autonomic nervous system plays a special role in energy homeostasis [65]. Under physiological conditions, SNS activation increases energy expenditure as a result of the breakdown of glycogen and the oxidation of glucose and fatty acids [66]. It has also been shown that ANS, thanks to the sympathetic innervation of white and brown adipose tissue, leads to the local release of norepinephrine, which promotes lipolysis in white adipose tissue and thermogenesis in brown adipose tissue [67].

**Figure 2:**
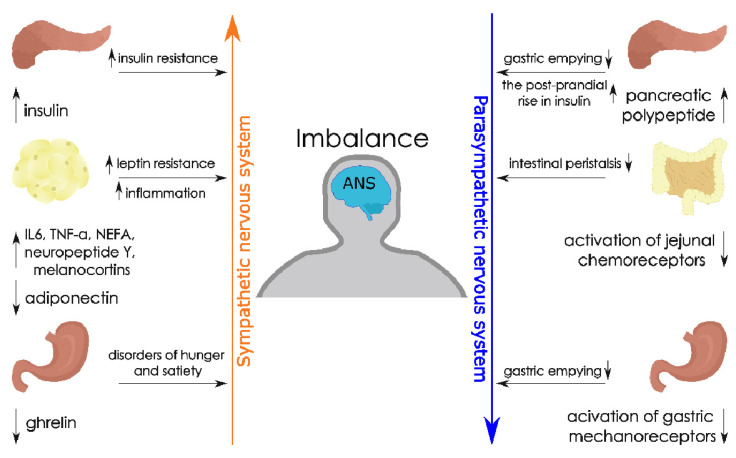
The possible imbalance of the sympathetic and parasympathetic branches of the autonomic nervous system, which may contribute to the dysfunction of the lymphatic system in the course of obesity. The figure prepared by Klaudia Antoniak on the basis of [45,49,53,70]. Abbreviations: ↑ — increase, ↓ — decrease, ANS (Autonomic Nervous System); TNF-α (tumor necrosis factor-α); IL-6 (Interleukin-6); NEFA (non-esterified free fatty acids).

Moreover, the SNS is responsible for the activation of the α2 adrenergic receptor in pancreatic β-cells and leads to the inhibition of insulin secretion [68]. Increased SNS activity may be induced by leptin in order to increase the metabolic activity of the liver and skeletal muscles, which should result in increased energy expenditure [57]. Hyper-insulinemia, low levels of ghrelin, and reduced sensitivity to adiponectin and leptin are indicated as potential mechanisms underlying excessive activation in obesity [69]. Moreover, cytokines such as TNF-α and IL-6, non-esterified free fatty acids (NEFA), neuropeptide Y, and melano-cortins have also been suggested as potential mediators of sympathetic activation in obesity [56,70]. On the other hand, stimulation of the vagus nerve, as a representative of the PNS, reduces the heart rate, stimulating intestinal peristalsis and the absorption of food from the gastrointestinal tract [71,72]. Studies on rats have shown that the efferent fibres of the vagus nerve have synapses on fat cells, and its activation improves insulin sensitivity and increases the uptake of energy substrates into adipose tissue [73,74]. Vagal afferent fibres have also been found to contribute to the regulation of satiety [75] and the release of intestinal hormones responsible for digestion [76]. Figure 2 shows a possible imbalance of the sympathetic and parasympathetic branches of the autonomic nervous system, which may contribute to the dysfunction of the lymphatic system in the course of obesity.

In several studies it has been shown that lymphatic vessels can be innervated by the autonomic nervous system [48,77,78]. In the work of Mignini et al., the presence of autonomic innervation of collectors and lymphatic vessels from the cervical, mesenteric and femoral areas in young and old people was examined. Both sympathetic and parasympathetic fibres were found in the lymphatic vessels. In addition, a reduction in the innervation of lymphatic vessels was observed in the elderly. The authors draw attention to the possible impairment of the lymphatic system function with age [77]. In a recent study, Cleypool et al., attempts to comprehensively locate sympathetic nerves in human lymph nodes [78]. A total number of 15 inguinal lymph nodes were resected from six donated human cadavers. The authors showed that all lymph nodes contained sympathetic nerves, both as para-vascular and discrete structures. In 15/15 lymph nodes, nerves were observed in their capsule, medulla and hilum, whereas only 13/15 lymph nodes contained nerves in their cortex. Human lymph nodes contain sympathetic nerves in their capsule, trabeculae, cortex, medulla and hilum, both as para-vascular and as discrete structures. The presence of discrete structures suggests neural regulation of structures other than blood vessels, which was further supported by the presence of varicosities in a portion of these nerves [78]. 

## 5. Lymphatic System—Physiology and Functions

The lymphatic system is distributed throughout the body and consists of lymphoid organs and lymphatic vessels [79,80]. Contrary to the circulatory system, it constitutes a one-way transport route from the extracellular space to the veins. In the lymphatic system, lymphoid organs have been classified as primary lymphoid organs and secondary lymphoid organs [80]. The primary lymphoid organs, including the thymus and bone marrow, are responsible for the production, maturation and selection of immune cells. From the primary lymphoid organs, lymphocytes enter the secondary lymphoid organs, or the spleen, lymph nodes, mucosa-associated lymphoid tissue (MALT), bronchus-associated lymphoid tissue (BALT) and gut-associated lymphoid tissue (GALT), where the cells of the immune system proliferate and mature [80,81]. On the other hand, the lymphatic vessel system is a unidirectional network which, based on the function as well as the size of the vessels, is divided into four parts [82,83]. Lymphatic capillaries, as the initial vessels, consist of a single LEC layer, connected directly to the interstitial tissue by “anchoring” in the surrounding connective tissue by means of fine filaments made of emilin and fibrillin [84,85]. The “anchoring” of the lymphatic vessel wall to the surrounding connective tissue is important in the presence of inflammation as well as lymphoedema [84]. In inflammation or lymphoedema, the taut filaments move the cells of the vessel wall apart, which allows passive opening of the intercellular junction and the flow of fluid into the lumen of the vessel [86]. In addition, LECs interlock with their protrusions, creating a typical intercellular junction: closure plates. In response to pressure differences, closure plates can open and close to regulate fluid reabsorption as well as the uptake of macromolecules and immune cells from the extracellular space into the lymphatic vessel [87,88]. Subsequently, the lymphatic capillaries converge into pre-collectors, vessels of larger diameter, located between the lymphatic capillaries and the collectors. As a result of their location, the function of the pre-collectors is partially similar to capillaries, and they are partially responsible for the resorption of the extracellular fluid. Other sections are responsible for the transport of lymph to the collectors [79]. Another part of the lymphatic vessel system are the collectors, which are proper transport vessels and, similarly to veins, have valves [89]. The distance between two valves is called a lymphangion, and the contraction of this section moves the lymph forward [79,90]. From the collectors, lymph is transferred to the main lymphatic vessels called lymphatic trunks [79]. The central lymphatic system is connected to the subclavian veins via the thoracic duct and right lymph duct, transporting interstitial fluid filtered by lymph nodes back to the circulatory system [87,91]. Lymph flow is conditioned by a combination of two factors: an active internal cycle of lymphangion contraction/relaxation and passive external compressive forces [92]. At rest, approximately one third of lymph transport in the lower extremities occurs as a result of external factors, while two thirds result from internal factors of the lymphatic vessel network [93]. In lymph transport, the internal factor is the mechanism of the contraction of the muscle layer, dependent mainly on the strength of the pressure gradient inside the lymphatic vessel [94]. External factors include skeletal muscle contractions, respiratory movements, changes in central venous pressure, pulsations of nearby arteries, and gravitational force [87,92,95,96,97,98]. Although lymphatic vessels were described almost 400 years ago [99], the molecular and cellular studies of the lymphatic system have mostly occurred the last two decades [100,101,102]. In recent years, the discovery of specific markers for the lymphatic system, such as vascular endothelial growth factor receptor 3 (VEGFR-3), podoplanin (gp38), Lymphatic Vessel Endothelial Receptor 1 (LYVE-1), and the lymphatic system-specific transcription factor Prospero Homeobox 1 (Prox-1) [84,102] allowed for the unequivocal identification of lymph vessels and isolation of LEC for in vitro analyses [103]. The role of the lymphatic system in controlling the flow of proteins, peptides, hormones, cytokines and other macromolecules to cells in all tissues has been described [79,81,104]. Moreover, it has been demonstrated that the proper functioning of the lymphatic system affects not only the balance of interstitial fluids and transport of immune cells [95,105], but also other aspects of homeostasis, such as fat absorption [106] and reverse cholesterol transport [107].

### 5.1. Lymphatic System—Fat Absorption and Cholesterol Transport

The intestinal lymphatic vessels are known as central vessels of the intestinal villi (lacteal vessels) [79]. Food fats are converted by enterocytes into chylomicrons—lipoproteins rich in triglycerides surrounded by proteins and cholesterol. Radiologic studies have shown that the central vessels of the intestinal villi have a spontaneous ability to contract, actively absorbing and transporting lipids processed by enterocytes into the systemic circulation [79,108]. Vasoconstriction occurs with the contraction of adjacent smooth muscle cells and is controlled by the ANS [109]. It has been shown that the central vessels of the intestinal villi control the absorption of lipids from food, which in turn may affect body weight [79,80].

Moreover, in peripheral tissues, the lymphatic system is considered to be the only way for lipoprotein to return to blood circulation [110]. The removal of cholesterol from the interstitial fluid by the lymphatic route is known as reverse cholesterol transport [111]. This is based on the transfer of cholesterol produced or accumulated in peripheral tissues to the liver or other tissues and organs. Reverse cholesterol transport is carried out by the HDL cholesterol fraction. The HDL particle in the form of preβ-HDL binds via its Apo-A1 receptor to the ABCA1 transporter present on the macrophage cell membrane [111]. As a result of this interaction, the cholesterol molecule is transported to the HDL particle, where it is esterified and transferred to the HDL core. The particle formed in this way is transferred to the liver through the lymphatic and blood vessels. In the liver, HDL is captured by SR-B1 receptors, and as a result of this process cholesterol esters are transferred to the hepatocyte cell without penetration of the HDL particle. Thus, the cycle of reverse cholesterol transport is completed [107,111]. So far, most of the research has been done on mice [107,112,113]. For example, in the studies by Martel et al. the authors measured reverse cholesterol transport by injection of [3H]-cholesterol-loaded macrophages into the peripheral tissues of mice to follow the mobilization of [3H]-cholesterol into plasma, liver and faeces [107]. Other studies show that the lymphatic system plays a special role in the transport and metabolism of cholesterol from peripheral tissues to the blood plasma, and that its impaired functioning leads to the accumulation of cholesterol in the tissues [112,113]. Moreover, due to the location of lymph capillaries in the outer membrane of atheromatous plaque, a key role of the presence of lymph capillaries in the drainage of local inflammatory cells and cytokines, and in protection against the development of atherosclerosis, has been suggested [107,114]. 

Moreover, transmission electron microscopy enabled the identification of lipid droplets in lymphatic endothelial cells, different reticulum cells, and macrophages, and the lipid droplet sizes, as well as their numbers and intercellular distances, increased after 10 weeks of high-fat diet feeding mice [115]. The results indicate that changes in the microarchitecture and increased accumulation of lipid droplets in stromal cells and macrophages influence the immunological function of the microarchitecture of the mesenteric lymph node [115].

### 5.2. Lymphatic System—Role in Regulating the Immune Response

The lymphatic system is not considered part of the immune system, but is critical to immunity [116,117,118,119,120,121,122,123,124,125]. The lymphatic system regulates the immune response by the transport of bacteria, foreign antigens, particulates, exosomes and immune cells to regional lymph nodes and lymphoid structures [118]. Non-specific immune cells, including dendritic cells (DCs), neutrophils, monocytes, and adaptive immune leukocytes such as T and B cells, use lymphatic vessels to migrate from tissues to lymph nodes [116,117,118,119,120,121,122]. 

Until recently, it was believed that the lymphatic system plays a passive role in regulating the immune response by transporting antigen-presenting cells and antigens to regional lymph nodes [123].

However, new findings suggest that the lymphatic system can control the immune response in a number of ways [116,117,122]. LECs have been shown to directly regulate the immune response by modulating the penetration of immune cells into lymph capillaries, presenting major histocompatibility complex antigens and modulating antigen presenting cells [120,121]. Moreover, it has been shown that the control of the immune response is possible through the presentation of the antigen in the lymph nodes mediated by LECs and lymph node stromal cells, as well as through the direct exit of lymphocytes from the lymph nodes [117]. In addition to the direct presentation of antigens, LECs can mediate molecular and cell transport or produce factors that have a large impact on the local environment [124]. As a result of this process, lymph vessels participate in the immune response in two ways; directly by controlling antigen/DCs transport to the draining lymph node, or indirectly by shaping the microenvironment of the lymph nodes [86,104]. Most of the lymphocytes and DC precursors migrate from the blood to the lymph nodes through separate post capillary venules called superior endothelial venules [125]. An alternative route for immune cells is provided by the lymphatic vessels, which do not transport from the blood, but directly from the interstitial fluid in adjacent tissues [104].

## 6. The Lymphatic System and Obesity

Recent studies have shown that obesity can significantly affect structural and functional changes in the lymphatic system [88,123]. Pathological lesions in the lymphatic system caused by obesity are partly a result of the accumulation of inflammatory cells around the lymphatic vessels [126,127]. In the course of obesity, decreased lymphatic vessel density, decreased lymphatic endothelial cell (LEC) proliferation, increased lymphatic permeability, and reduced lymphatic contractility are observed [85,122,126,127,128]. In the study by Greene et al., particular attention was paid to the disturbance of the transport function of the lymphatic system, and thus the presence of lymphatic stasis [129]. The impaired removal of excess macromolecules such as lipids and proteins from the interstitial space, and the impaired transport of immune complexes, T cells or Langerhans cells have been shown. Additionally, the research pointed to the possible malfunction of antigen-presenting cells and the possible occurrence of abnormalities in the structure of the lymph nodes [100,129]. In a study by Nitti et al., a mouse model of diet-induced obesity was used to identify putative cellular mechanisms of obesity-induced lymphatic dysfunction and determine whether there is a correlation between these deleterious effects and increasing weight gain [91]. The authors showed a positive relationship between weight gain and declining function of the lymphatic system, also characterized by reduced lymphatic vessel density and reduced lymphatic vessel pumping frequency [91]. 

In other studies, a decreased flow of dendritic cells from the tissues to the lymph nodes was demonstrated [122,130]. In addition, the characterization of lymphatic vascular development and our understanding of this vasculature’s role in pathophysiological conditions has greatly improved in recent years, changing conventional views about the roles of the lymphatic vasculature in health and disease [131].

Conversely dysfunction in the lymphatic system may also be involved in the pathogenesis of obesity [87,90,128,132,133]. Data linking lymphatic dysfunction with obesity were initially provided by mouse models with lymphatic defect [90,128,132]. For example, Chy mice, a mouse model of lymphedema due to heterozygous inactivating mutations in VEGFR-3, exhibit abnormal subcutaneous fat deposition predominantly in the edematous subcutaneous adipose layer adjacent to the dysfunctional hypoplastic lymphatic vessels [90]. In another study in Prox1+/− mice, compromised lymphatic vascular integrity caused subtle leakage of lymph [128]. Free fatty acids containing lymph accumulated in the nearby tissues, particularly in the visceral area, where it induced de novo differentiation of fat cell precursors, fat cell hypertrophy and eventually adipocyte proliferation. With age, Prox1+/− mice became progressively obese [128]. The results of the experimental research are supported by clinical reports that documented the spontaneous development of lymphedema in obese patients [87,101]. In patients with secondary lymphoedema caused by a disorder of the lymphatic system, clear adipose tissue hypertrophy and accompanying tissue oedema and fibrosis were observed [87]. In turn, Varaliova et al. made an attempt to assess the relationship between lymphatic flow and the regulation of adipose tissue metabolism in women with gynoid obesity [101]. It has been shown that reduced lymphatic flow can change the composition of the interstitial fluid, thereby affecting the adipocyte environment. In addition, women with reduced lymphatic flow showed a lower lipolysis index and a reduced response to adrenergic stimuli of the femoral adipose tissue. The above observations may indicate that disorders of the lymphatic system function may be the reason for the reduction of adipose tissue loss in gynoid obesity [101]. Other reports indicate that any minor damage to the lymphatic system activates adipose tissue differentiation genes and leads to adipose tissue hypertrophy and proliferation [133]. These studies suggest lymphatic dysfunction causes the activation of adipocytes and the accumulation of adipose tissue, and thus may promote obesity [128,132,133].

## 7. Dysfunction of the Lymphatic System: Pro- and Anti-Lymphangiogenic Factors in Obesity

The lymphatic system plays a key role in regulating the inflammatory response, influencing the drainage of extravasated fluid, the transport of inflammatory mediators and immune cells [82]. It is now well known that lymphatic vessels are highly dynamic structures that undergo both morphological and functional changes under pathological conditions [131,134,135,136,137,138,139].

Inflammation can lead to an increase in microvascular permeability and in the volume of interstitial fluid [134]. Increasing this volume causes stretching of the lymphatic vessel wall, which contributes to increasing the frequency and/or force of lymphangion contractions [134,135,136]. The inflammatory environment induces changes of gene expression in LECs and leads to profound proliferative expansion of the lymphatic network in both inflamed tissue and lymph nodes [137,140,141,142,143,144,145,146]. Research results indicate that inflammation in lymphangio-genesis is regulated by the expression of both pro- and anti-lymphangiogenic factors, which are produced by various immune cells, depending on the inflammatory stimulus [123,147,148,149]. In inflamed tissue on transgenic mice, lymphangio-genesis has been shown to be mediated by macrophage influx caused by the expression of vascular endothelial growth factor A (VEGF-A) and vascular endothelial growth factor C (VEGF-C) [149]. In the studies of Wada et al., VEGF-A levels detected in human subjects were significantly and more strongly correlated with the body mass index (BMI) and waist circumference than VEGF-C [150]. Conversely, VEGF-C levels were significantly and more closely correlated with metabolic (e.g., fasting plasma glucose, HbA1c, insulin, and the homeostasis model assessment of insulin resistance) and lipid parameters (e.g., triglycerides, total cholesterol (TC), low-density-lipoprotein cholesterol (LDL-C), and non-high-density-lipoprotein cholesterol (non-HDL-C) than VEGF-A. Stepwise regression analyses revealed that independent determinants of VEGF-A were BMI and age, whereas strong independent determinants of VEGF-C were age, triglycerides, and non-HDL-C. The authors in the applications state that that circulating levels of VEGF-C are closely associated with dyslipidemia and atherosclerosis. However, endogenous VEGF-C in human, and its relationships with cardiovascular lymphangiogenic activity, are unclear [150].

Other studies have shown that IL-17 induces VEGF-D expression, thereby triggering an indirect induction of lymphangio-genesis [151] and IL-8 promotes lymphangio-genesis in animal models of lymphoedema [152]. On the other hand, both in vitro and in vivo studies [153,154,155] have shown that interferon-γ (IFN-γ), TGF-β, IL-4 and IL-13, as well as higher levels of adiponectin are factors that inhibit lymphangio-genesis. For example, Savetsky et al., in studies on human skin lymphatic endothelial cells (hLEC) and adult male C57/BL6 mice (10–12) weeks, showed that IL-4 and IL-13 administration in physiologic doses have profound anti-lymphangiogenic effects and potently impair LEC survival, proliferation, migration, and tubule vessel formation [153].

**Figure 3:**
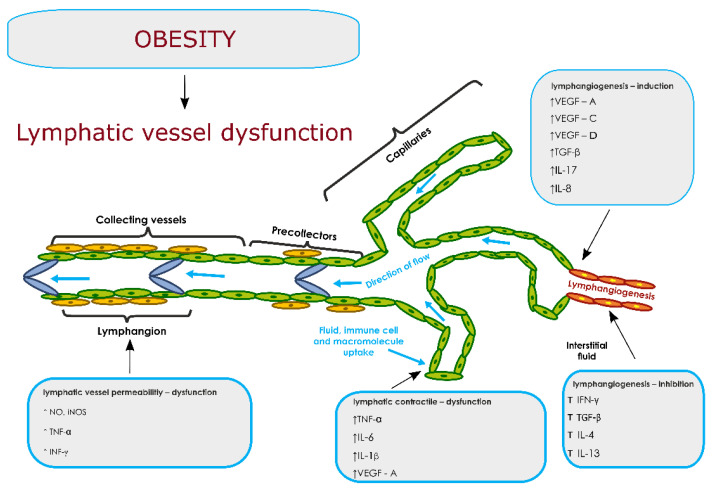
The possible influence of pro- and anti-lymphangiogenic factors in the dysfunction of the lymphatic system in the course of obesity. The figure prepared by Klaudia Antoniak on the basis of literature chapter 7. Abbreviations: ↑ — increase, ↓ — decrease, TNF-α (tumor necrosis factor-α); IL-1β (Interleukin-1β); IL-6 (Interleukin-6); iNOS (induced nitric oxide synthase); NO (nitric oxide); IL-10 (Interleukin-10); IL-1 (Interleukin-1); IL-8 (Interleukin-8); IFN-γ (Interferon-γ); IL-4 (Interleukin-4); IL-13 (Interleukin-13); IL-17 (Interleukin-17); TGF-β (transforming growth factor-β); VEGF-A (vascular endothelial growth factor A); VEGF-C (vascular endothelial growth factor C); VEGF-D (vascular endothelial growth factor D).

In turn, Avraham et al., demonstrated that TGF-β1 expression is increased in the lymphoedematous limbs of patients as compared with matched biopsy specimens from their unaffected extremity. Using a mouse tail model, they demonstrated that systemic or local inhibition of TGF-β1 markedly improves lymphangio-genesis during wound repair, is associated with decreased expression of IL-4 and IL-13, and significantly decreases chronic inflammation and tissue fibrosis [155]. Other researchers in experimental studies in mice have demonstrated the effect of adiponectin in inhibiting lymphangio-genesis [156]. A mouse model of lymphedema was created via ablation of the tail surface lymphatic network. Adiponectin-knockout mice showed the greater diameter of the injured tail compared with wild-type mice, which was associated with lower numbers of lymphatic endothelial cells (LECs). Systemic delivery of adiponectin reduced the thickness of the injured tail and enhanced LEC formation in wild-type and adiponectin-knockout mice. Adiponectin administration also improved the oedema of injured tails in obese KKAy mice [156]. Figure 3 shows the influence of pro- and anti-lymphangiogenic factors in the dysfunction of the lymphatic system in the course of obesity

### 7.1. Lymphatic System Dysfunctions—Permeability of Lymphatic Vessels

Inflammation can lead to increased permeability of lymphatic vessels and increase the volume of interstitial fluid [157,158,159,160]. Increasing its volume causes stretching of the lymphatic vessel wall, which contributes to increasing the frequency and/or force of lymphangion contractions and to the increase the lymphangio-motoric activity to prevent oedema development [134,135]. In an in vitro study (endothelial cells were obtained from mesenteric collecting lymphatics of Sprague-Dawley rats), the authors evaluated the integrity of the lymphatic barrier in response to inflammatory stimuli commonly associated with increased blood endothelial permeability [157]. They utilized in vitro assays of the lymphatic endothelial cell (LEC) monolayer barrier function after treatment with different inflammatory cytokines and signalling molecules including TNF-α, IL-6, IL-1β, IFN-γ and LPS. Moderate increases in an index of monolayer barrier dysfunction were noted with all treatments (20–60% increase) except IFN-γ, which caused a greater than 2.5-fold increase [157]. In addition, researchers indicate that excessive permeability of lymphatic vessels is undesirable in maintaining fluid and macromolecule homeostasis, leading to potential tissue injury [157].

In a study by Ciffarelli et al., an attempt was made to assess the relationship between the level of lymphatic permeability and the time of lipid transport in the intestinal lactic vessels [159]. For this purpose, male and female mice divided into two groups were included in the study. The test group consisted of mice with an induced deletion of CD36 (fatty acid transporter) in the LEC (Cd36ΔLEC), the control group consisted of wild-type (WT) mice. In both groups, a high-fat diet was used for 12 weeks, body weight was determined, an oral glucose load test was performed, and the function of lymphatic vessels was analysed. It has been shown that, along with the increased permeability of the intestinal lactic vessels (Cd36ΔLEC mice), slower transport of absorbed lipids is observed, and in addition increased VAT accumulation, increased inflammation and decreased glucose tolerance were observed [159].

Dysfunction of permeability of lymphatic vessels caused by histamine and thrombin was presented too in a study on micro-lymphatic endothelial cells of skin origin [160]. However, only a few studies concern in vivo assessment of permeability of lymphatic vessels. The results of these studies indicate the role of VEGF-A as an important factor increasing vascular permeability, while overexpression of VEGF-A is observed both in cancer patients and in patients with chronic inflammation [161].

### 7.2. Lymphatic System Dysfunctions—Lymphatic Contractility

Similar to the smooth muscle activity of blood vessels, the contractile activity of the muscles of lymphatic vessels shows basic muscle tone, the muscle’s response to pressure changes [161], and is also modulated by various biological, neuro-modulatory, vasomotor and mechanical factors [95]. Additionally, ample evidence suggests that lymphatic contractility is dependent on the autonomic nervous system [48,162,163]. Research indicates that ANS cholinergic and adrenergic activity may alter the frequency and strength of lymphatic contractions [162].

It has been shown that physiological production of nitric oxide in lymphatic vessels can support LEC barrier integrity, promote LEC proliferation and regulate lymphangio-motoric activity [164]. However, in inflammation, the level of NO is elevated due to a higher expression of inducible nitric oxide synthase, which is associated with a limitation of the LEC barrier function and a decrease in contractility of lymphatic vessels, thus reducing their lymphangio-motoric activity [165]. In addition, in the study by Muthuchamy et al., a reduction in mesenteric lymphatic vessel lymphangio-motoric activity under the influence of TNF-α and IFN-γ, which are potent stimulators of NO secretion, was observed [166].

Studies have shown that inflammatory mediators such as prostaglandins and histamine are among the biological factors inhibiting lymphangio-motoric activity [166]. On the other hand, the activity enhancing it is primarily attributed to VEGF-C [166,167,168]. Interestingly, the results of the research show that the lymphatic system is also influenced by known standard therapies used in the treatment of inflammatory diseases, including rheumatoid arthritis, where TNF-α blocking contributes to increased lymphangio-genesis in inflamed tissues [169,170]. It is also important that cytokines, chemokines and growth factors often have pleiotropic effects, making it difficult to distinguish between direct and indirect mechanisms [133,158]. The wide range of inflammatory mediators as well as their ambiguous effects on the lymphatic system result in a very complex network of signals that is still not fully understood [103,133,168]. 

## 8. Manual Lymphatic Drainage as a New Therapeutic Option Supporting the Functions of the Lymphatic System in the Course of Obesity

Manual lymphatic drainage (MLD) is one of the physiotherapeutic methods that was first described in the 1930s by Emil Vodder [171]. Manual lymphatic drainage is widely used in the treatment of lymphoedema, venous oedema, and lipoedema [172,173,174,175,176,177]. The aim of MLD is primarily to increase lymph flow, as well as restore the balance between the load on the lymphatic system and its transport capacity [176,178].

The transport capacity of the lymphatic system was assessed in the study of de Godoy et al. [174]. Six patients with lymphoedema in the lower extremity were enrolled in the study. The aim of the study was to evaluate the transport of radiotracers in lymphatic collectors using lymphoscintigraphy before, during and after MLD therapy. The study proved that MLD therapy improves the transport of radiotracers in lymphatic collectors [174].

In addition, an exploratory pilot study was conducted to determine the possibility of assessing the effectiveness of MLD using the fluorescence lymphography method. The study involved 12 healthy subjects and 10 patients with lymphoedema. Subjects were given one MLD therapy, and the contractility and transport capacity of the lymphatic vessels were assessed by fluorescence lymphography before and after MLD therapy. The study showed increased contractility of lymphatic vessels in a group of healthy subjects as well as in a group of patients with lymphoedema [175].

Similar results were obtained in the study performed by Belgrado et al., in which 30 healthy participants underwent a 15-min MLD therapy of the upper extremity. Fluorescence lymphography was used to assess the contractility and transport capacity of the lymphatic vessels in real time. The study also showed an increase in lymph transport in lymphatic vessels during MLD therapy [179].

There are also reports suggesting a positive effect of MLD on the treatment of women with infertility [180], as well as people with rosacea accompanied by chronic lymphoedema of the face [181]. The search for therapeutic interventions aimed at supporting the treatment of obesity, including the reduction of comorbid chronic inflammation, has been the subject of the work only of a few researchers, including our team [101,182,183,184,185]. For example, a study by Arngrim et al., investigated subcutaneous adipose tissue lymphatic drainage of macromolecules in lean and obese subjects and, furthermore, evaluated whether adipose tissue lymphatic drainage may change in parallel with adipose tissue blood flow [182]. Twelve patients were examined, including six slim healthy men, BMI = 22.3 ± 1.2 and 6 obese, BMI = 35.7 ± 4.5, but otherwise healthy men with normal glucose tolerance (determined by an oral glucose tolerance test). Whole body adipose tissue was determined by dual energy X-ray absorptiometry, and the thickness of the periarticular adipose tissue of the skin was measured with the Harpenden Skinfold Caliper. It has been shown that a significant increase in lymphatic drainage was seen after the glucose load in the lean subjects. In the obese subjects, lymphatic drainage remained constant throughout the study and was significantly lower compared to the lean subjects. The authors suggest that the obtained results indicate a reduced ability to remove macromolecules from the interstitial space through the lymphatic system in obese subjects. This may cause a high local production of pro-inflammatory cytokines and as a consequence the development of obesity-related inflammation in hypertrophic adipose tissue [182]. Bertelli et al., investigated the effect of postural drainage and manual lymphatic drainage techniques on lower limb oedema in extremely obese women undergoing bariatric surgery. The authors suggest that both techniques could be used to help reduce lower limb oedema among this population. Nevertheless, the best results were obtained with MLD [183]. Our initial research indicated the potential benefits of the application of MLD in improving biochemical parameters, including a reduction in the inflammation and improvement in the quality of life in patients with overweight and obesity class 2 [184,185]. However, further studies on a larger number of patients, both overweight and obese, are needed to thoroughly investigate the effectiveness of manual lymphatic drainage in the course of obesity. Perhaps manual lymphatic drainage will be recognized as a non-invasive and effective method supporting the treatment of obesity, which may lead to the prevention of many diseases.

## 9. Conclusions

In recent years, it has been proven that adipose tissue, the excess of which is present in obesity, is an important endocrine organ that synthesizes and secretes many biologically active substances. As previously shown, there are various types of cell in adipose tissue, both adipocytes and fibroblasts, macrophages and lymphocytes, which to a greater or lesser extent participate in the secretory function of WAT. Moreover, both types of WAT, subcutaneous and visceral, differ in metabolic activity and the ability to secrete adipokines. They act within adipose tissue and distant organs and tissues, and their overproduction may lead to low-grade inflammation in various disease entities, including obesity. Moreover, considering the closely related localization and mutual communication between adipose tissue, and the lymphatic system, it is likely that chronic inflammation affects the dysfunction of the lymphatic system in the course of obesity. Therefore, it is important to seek therapeutic interventions to support the treatment of obesity, including the reduction of the comorbid chronic inflammation. Manual lymphatic drainage may be one of the promising forms of therapy improving the function of the lymphatic system in patients with abnormal body weight.

## Figures and Tables

**Table 1:** Adipose tissue in obesity—selected metabolic effects.

Cell Type	Synthesis of Factors	Metabolic Effects	Source
↑ Macrophages Polarization of M2 macrophages towards M1 inflammatory macrophages	↑ TNF-α, IL-1β, IL-6, IL-12, IL-23 ↑ iNOS activation and ↑ NO ↓ IL-10	↑ inflammation	[13,14,15,16,17,20]
		↑ insulin resistance	
		↑ risk of atherosclerosis development	
		↑ risk of hypertension development	
↑ Neutrophils	↑ TNF-α, MCP-1, IL-1β	↑ macrophage infiltration	[20,21,22]
		↑ insulin resistance	
↑ Mast cells	↑ IL-1, IL-3, IL-5, IL-6 IL-8, TNF-α, histamines, heparins, tryptases, chymases	↑ macrophage infiltration remodeling of adipose tissue	[23,24]
↓ Treg	↓ IL-1β, IL-6, IL-8, TNF-α, MIP-1α	↑ inflammation	[27,28,29]
		↑ insulin resistance	
		↑ M1 macrophage infiltration	
		↓ glucose tolerance	
↑ CD4+ Th1	↑ IFN-γ	↑ insulin resistance	[27,29,30]
		↑ risk of atherosclerosis development	
		↑ risk of hypertension development	
		change in phenotype of M2 macrophages towards M1 macrophages	
↓ CD4+ Th2	↓ IL-4, IL-5, IL-13, IL-10	↓ glucose tolerance	[32,33,34]
↑ CD4+ Th17	↑ TNF-α and IL-17	↑ insulin resistance	[32,33,34]
		↑ risk of atherosclerosis development	
		↑ risk of hypertension development	
		change in phenotype of M2 macrophages towards M1 macrophages	
↑ T CD8+	↑ TNF- α, IL-2, IFN- γ and RANTES chemokines	↑ insulin resistance	[32,33,34]
↓ B Cells	↑ IL-6	activation of pro-inflammatory CD4+ and CD8+ lymphocytes	[34,35,36,37]
	↓ IL-10	change in phenotype of M2 macrophages towards M1 macrophages	
↑ Saturated Fatty Acids—as a metabolites odd-chain saturated fatty acids even-chain saturated fatty acids	↑ MCP-1 and TNF-α	change in phenotype of M2 macrophages towards M1 macrophages	[40]
	↑ resistin	odd-chain saturated fatty acids are associated with a favorable adipokine profile, whereas even-chain saturated fatty acids are associated with an unfavorable profile.	[39]
	↓ adiponectin		
	↑ visfatin		
	↓ leptin and PAI-1		

Abbreviations: ↑ — increase, ↓ — decrease, TNF-α (tumor necrosis factor-α); IL-1β (Interleukin-1β); IL-6 (Interleukin-6); IL-12 (Interleukin-12); IL-23 (Interleukin-23); iNOS (induced nitric oxide synthase); NO (nitric oxide); IL-10 (Interleukin-10); MCP-1 (Monocyte Chemoattractant Protein-1); IL-1(Interleukin-1); IL-3(Interleukin-3); IL-5 (Interleukin-5); IL-8 (Interleukin-8); Treg (regulatory T cells); MIP-1α (macrophage inflammatory protein-1α); Th1 (T-helper type 1); IFN-γ (Interferon-γ); Th2 (T-helper type 2); IL-4 (Interleukin-4); IL-13(Interleukin-13); Th17 (T-helper type 17); IL-17 (Interleukin-17); IL-2(Interleukin-2); plasminogen activator inhibitor-1 (PAI-1).

## Abbreviations

WHO	World Health Organization
ANS	autonomic nervous system
BALT	Bronchus-associated lymphoid tissue
BAT	Brown adipose tissue
BMI	body mass index
CGRP	calcitonin gene-related peptide
DCs	dendritic cells
EEG	electroencephalogram
GALT	Gut-associated lymphoid tissue
gp38	podoplanin
HRV	heart rate variability
IFN-γ	Interferon-γ
IL-1	Interleukin-1
IL-10	Interleukin-10
IL-12	Interleukin-12
IL-13	Interleukin-13
IL-17	Interleukin-17
IL-1β	Interleukin-1β
IL-2	Interleukin-2
IL-23	Interleukin-23
IL-3	Interleukin-3
IL-4	Interleukin-4
IL-5	Interleukin-5
IL-8	Interleukin-8
iNOS	induced nitric oxide synthase
IR	insulin resistance
JNK	c-Jun N-terminal kinases
LEC	lymphatic endothelial cell
LYVE-1	Lymphatic Vessel Endothelial Receptor 1
MALT	mucosa-associated lymphoid tissue
MIP-1α	macrophage inflammatory protein-1α
MLD	manual lymphatic drainage
NA-SR	noradrenaline spillover rate
NEFA	Non-esterified free fatty acids
NF-κB	nuclear factor kappa-light-chain-enhancer of activated B cells
NK	natural killers
NO	nitric oxide
PAI-1	plasminogen activator inhibitor-1
PNS	parasympathetic nervous system
Prox-1	Prospero Homeobox 1
RBP-4	retinol-binding protein
SNA	sympathetic nerve activity
SNS	sympathetic nervous system
SP	substance P
SVF	Stromal vascular fraction
T2DM	Type 2 diabetes
TGF-β	Transforming growth factor-β
Th1	T-helper-type 1
Th17	T-helper-type 17
Th2	T-helper-type 2
TNF-α	Tumor necrosis factor-α
IL-6	Interleukin-6
MCP-1	Monocyte Chemoattractant Protein-1
Treg	regulatory T cells
VAT	Visceral adipose tissue
VEGF-A	vascular endothelial growth factor A
VEGF-C	vascular endothelial growth factor C
VEGF-D	vascular endothelial growth factor D
VEGFR-3	vascular endothelial growth factor receptor 3
VIP	vasoactive intestinal peptide
WAT	White adipose tissue
